# Supplementary material for: Complex‐centric proteome profiling by SEC‐SWATH‐MS
Source: Mol Syst Biol. 2019 Jan 14;15(1):e8438. doi: 10.15252/msb.20188438 (PMC6346213; doi:10.15252/msb.20188438)
Supplement: Supplementary file 8 — Dataset EV7 [file MSB-15-e8438-s008.zip › feature_plots_string/Q8TCX1.pdf]

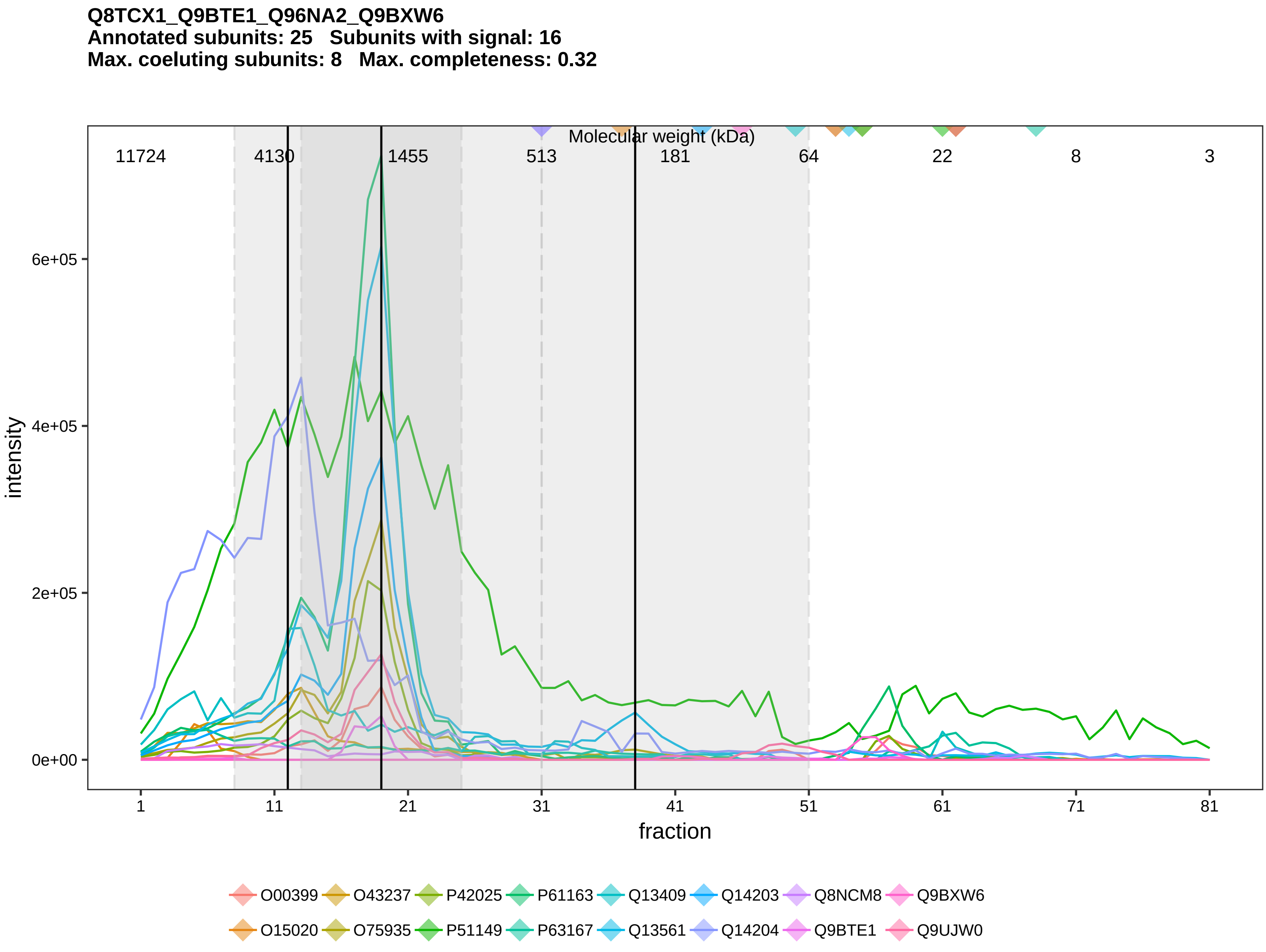
Q8TCX1_Q9BTE1_Q96NA2_Q9BXW6
Annotated subunits: 25 Subunits with signal: 16
Max. coeluting subunits: 8 Max. completeness: 0.32
Molecular weight (kDa)
11724
4130
1455
513
181
64
22
8
3
intensity
6e+05
4e+05
2e+05
0e+00
1
11
21
31
41
51
61
71
81
fraction
O00399
O43237
P42025
P61163
Q13409
Q14203
Q8NCM8
Q9BXW6
O15020
O75935
P51149
P63167
Q13561
Q14204
Q9BTE1
Q9UJW0
